# Mixed reality as a novel tool for diagnostic and surgical navigation in orthopaedics

**DOI:** 10.1007/s11548-020-02302-z

**Published:** 2021-02-08

**Authors:** Andrea Teatini, Rahul P. Kumar, Ole Jakob Elle, Ola Wiig

**Affiliations:** 1 The Intervention Centre, Oslo University Hospital, Oslo, Norway; 2 Department of Informatics, University of Oslo, Oslo, Norway; 3 Department of Orthopaedic Surgery, Oslo University Hospital, Oslo, Norway

**Keywords:** Image-guided treatment, Image-guided diagnosis, Surgical navigation, Orthopaedics, Orthopaedic surgery, Augmented reality, Mixed reality, Holographic visualisation

## Abstract

**Purpose:**

This study presents a novel surgical navigation tool developed in mixed reality environment for orthopaedic surgery. Joint and skeletal deformities affect all age groups and greatly reduce the range of motion of the joints. These deformities are notoriously difficult to diagnose and to correct through surgery.

**Method:**

We have developed a surgical tool which integrates surgical instrument tracking and augmented reality through a head mounted display. This allows the surgeon to visualise bones with the illusion of possessing “X-ray” vision. The studies presented below aim to assess the accuracy of the surgical navigation tool in tracking a location at the tip of the surgical instrument in holographic space.

**Results:**

Results show that the average accuracy provided by the navigation tool is around 8 mm, and qualitative assessment by the orthopaedic surgeons provided positive feedback in terms of the capabilities for diagnostic use.

**Conclusions:**

More improvements are necessary for the navigation tool to be accurate enough for surgical applications, however, this new tool has the potential to improve diagnostic accuracy and allow for safer and more precise surgeries, as well as provide for better learning conditions for orthopaedic surgeons in training.

## Introduction

A large number of patients suffer from different joint and skeletal diseases. These can be both congenital and acquired and affect all age groups. Perthes disease, hip dysplasia and epiphysiolysis capitis femoris are examples of such pathological conditions. These conditions cause hip, knee and groin pain, and reduced range of motion of the joint. These can lead to a limping gait, problems with activity of daily life and reduced quality of life. However, skeletal and joint conditions are notoriously difficult to diagnose and treat. Diagnosis of orthopaedic conditions are largely dependent on the experience of the clinician and the outcome of the surgery is dependent on the surgeon’s ability to understand the pathological anatomy through both diagnostic testing as well as his or her practical surgical abilities.

Clinicians largely base their diagnosis on clinical experience and medical imaging, such as conventional X-ray combined with either computed tomography (CT) or magnetic resonance imaging (MRI). However, these images are only displayed as static images, visualised in 2D screens. From 2D slices of the medical images, through segmentation processes, 3D models can be reconstructed to visualise the regions of interest. However, these 3D medical reconstructions are still static volume renderings and have to be visualised on a 2D screen.

To perform diagnostics and surgical outcome evaluation, there is a need to visualise real-time motion of patient specific model reconstructions, according to the motion of the patient. During surgery or post-operatively, fluoroscopic imaging is currently used to assess the progress and outcome of the procedure. This leads to an increase in radiation exposure to both the patient and the Operation Room (OR) staff. Surgical navigation technologies have been developed, using surgical instrument tracking technologies, such as NDI$^\circledR$’s Polaris Spectra optical tracking (Northern Digital Inc. (NDI), Waterloo, Ontario, Canada), to reduce the need of intraoperative imaging. These systems have been used for several years in multiple fields of application [1–3]. They allow lowering unnecessary radiation while also improving surgical safety and accuracy.

Most of the navigation solutions available on the market, however, limit the visualisation to an external 2D monitor, which reduces the clinician’s ability to see the patient’s movements and the model’s motion simultaneously in the same space. Moreover, in surgical scenarios, interaction within the OR with the models on the 2D monitors needs to be performed by other operators (which can result in time consuming and tiresome work for the surgeon), unless sterile touchscreen devices or drapes are available close to the surgeon. Furthermore, visualisation of CT or MRI imaging on 2D screens has been demonstrated to require more time for surgical diagnostics and planning compared to 3D model visualisation as holographic images [4].

Mixed Reality (MR) offers solution to the previously described problems by allowing to visualise virtual patient-specific 3D models that can interact with the physical world of the user. Compared to virtual reality, where the user can only see virtual structures, and augmented reality, where the user can see the virtual structures as an overlay to the physical world, the user can visualise both virtual and physical objects in the same space and interact with them without the need to touch the monitors. This allows interaction with 3D models to be performed directly by the surgeons, in sterile conditions, during an operation [5].

In order to improve the safety and usability of MR-based surgical navigation tools, we incorporated surgical instrument tracking technologies (such as optical and electromagnetic tracking) with MR attained through Microsoft’s$^\circledR$ HoloLens headset because it has already shown its efficacy for similar purposes in other projects [6]. This tool aims to provide better understanding of the pathological anatomy compared to conventional diagnostic tools by showing dynamic holograms which follow the movements of the patient and surgical instrument navigation. The tool also offers ease-of-use for clinicians, thanks to the user’s illusion of having “X-ray” vision when the models are registered to the patient’s anatomy. Through simple hand gestures in the air, the user is able to rotate, translate and scale 3D models on top of the real world. HoloLens does not need external wires or cameras and the user can move freely and untethered.

This study aims to evaluate the accuracy of this mixed reality tool for clinical use for orthopaedic hip and pelvic diagnostics and surgery. However, the tool accuracy assessment holds for navigation in any surgical procedures (e.g. open and laparoscopic abdominal surgery, aortic valve placement, hip replacement surgery). The registration methods implemented in this study aim to provide surgeons the ability to perform a real-time examination of the hip joint and pelvis allows them to observe the interaction between the femoral head and the acetabulum, thus being able to accurately diagnose impingement and other anomalies in the hip joint.

The registration methods described in this study could also, in the future, be used for surgical applications for specialised tasks to tackle pathological conditions. An example could be the MR visualisation of osteotomy planes: with this application, the osteotomy planes can be applied to the holographic model of the hip, so that the surgeon is able to see where to cut the bone, and as well as to place the initial skin incision. The incision can be made smaller, as the surgeon knows exactly where to perform the osteotomies, as he/she will not have to expose larger parts of the femur or pelvis in order to obtain a precise understanding of the anatomy.

## Materials and methods

**Fig. 1:**
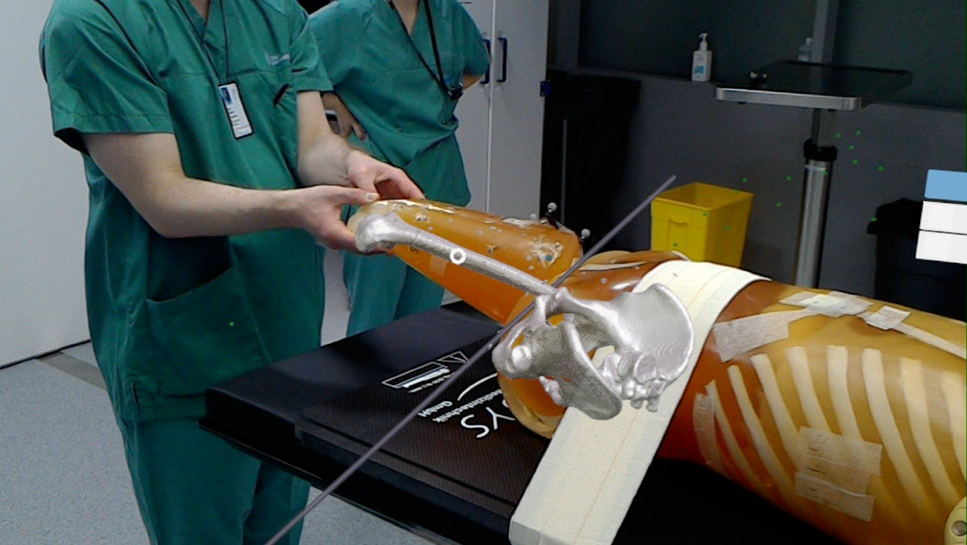
Experiment with the Patient phantom, seen with the HoloLens as augmented reality while the surgeon manipulates the limb. The grey line is the edge of the Polaris measurement volume (frustrum) visualised in HoloLens. This snapshot from the HoloLens video is taken from the left eye view, hence, does not fully reproduce the accuracy of the system (which, rendered in 3D view, is much more accurate as described in “Patient phantom” section)

The tool developed in this study uses 3D holograms which are reconstructed via segmentation processes from the patient’s CT or MRI scans, depending on the surgical needs (for example, cartilage may require MRI rather than CT). The 3D models are then imported into holographic volumes in the HoloLens. A Polaris Spectra, NDI$^\circledR$, was used as means of optical tracking for both the HoloLens camera and the pointer used for evaluation (NDI$^\circledR$ 8700340). The Optical Tracking System (OTS) was used to compute image-to-patient registration using the pointer. Using an optically tracked reference plate (NDI$^\circledR$’s reference frame 8700449) strapped to the patient’s limb, the holographic model can be seen moving together with movement of the patient in real-time. The use of the instrument tracking technologies allows the surgeon/clinician to visualise dynamic updates of the location and orientation of the femoral bone, and, for patients with hip pathologies, tracking allows the surgeon to see and understand the pathological hip-femoral head impingement untethered and within a large measurement volume (as opposed to other solutions that allow up to a maximum of 20 cm [5]). The study evaluates the accuracy in tracking a location at the tip of an instrument in holographic space of the navigation tool through two different phantom studies: the first experiment evaluates the accuracy through a custom-built optical verification phantom, designed to fit American Society for Testing and Materials (ASTM$^\circledR$) standard F2554-10 for accuracy evaluation (more information in [7]). The second experiment evaluates the accuracy of the navigation tool in a more clinical scenario, using a whole-body CT patient phantom (PBU-50 Kyoto Kagaku Co.$^\circledR$), which is a full patient-body sized anthropomorphic phantom with movable and detachable joints, built with synthetic adult-sized bones. Furthermore, the accuracy was also evaluated qualitatively by an orthopaedic surgeon, who used the navigation tool, performing testing on the patient phantom while moving the phantom’s inferior limb mimicking the movements performed on the patient during pre- and post-operative diagnostic analysis (as shown in Fig. 1).

The navigation tool was developed and designed through a combination of computer-aided design modelling, computer vision algorithms and instrument tracking technologies, which are described in the following paragraphs.

### Image-to-patient registration

Image-to-patient registration computes the transformation from the image space to the position of the patient. In this study, the image space is represented by a on rails Siemens SOMATOM CT scanner and the position of the patient is on the operation table prepared for surgery or in the biomechanical diagnostics laboratory. The most common approaches in the literature for image-to-patient registration is rigid transformations based on landmarks [3, 8, 9].

**Fig. 2:**
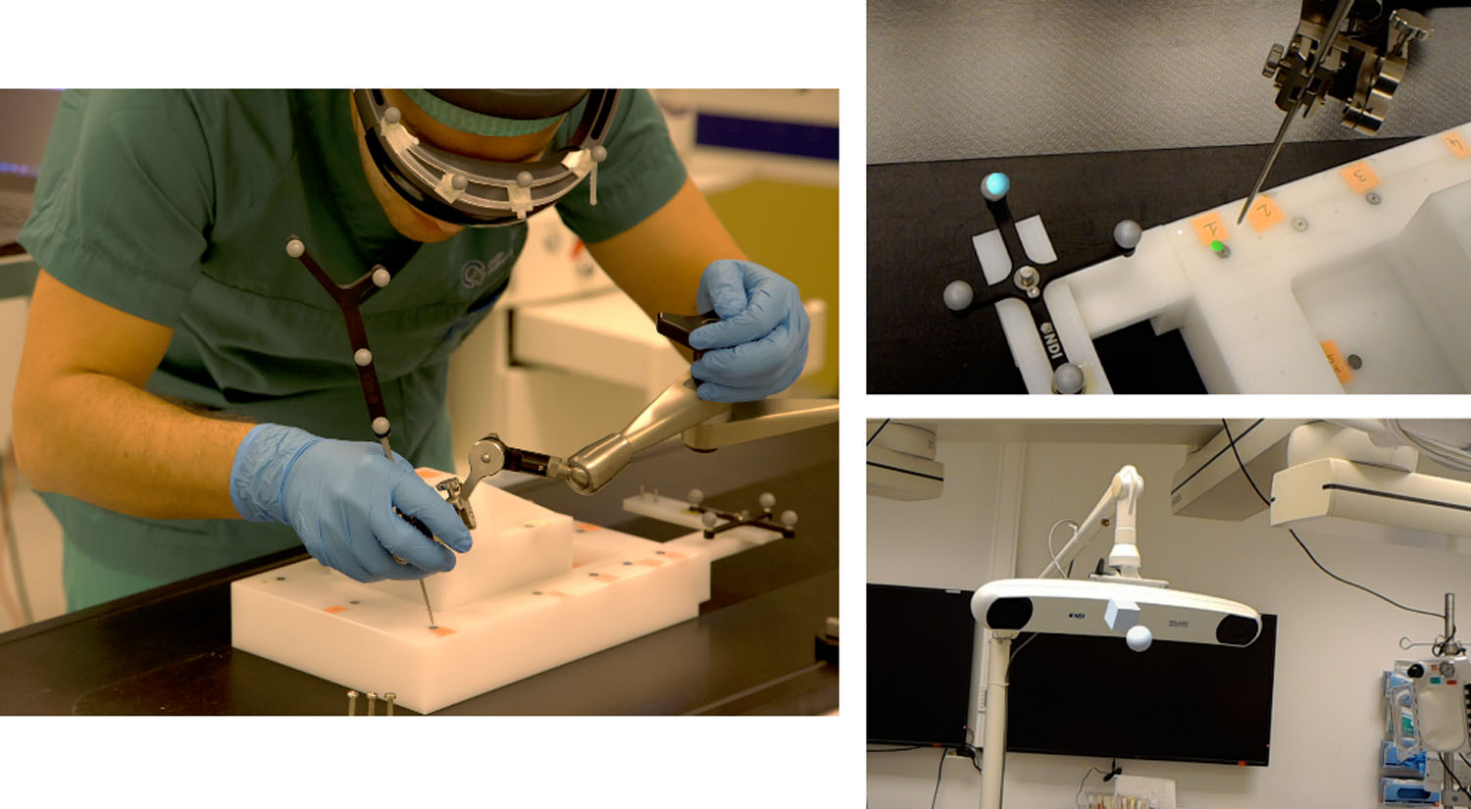
Evaluation experiment through the Validation phantom. The left image shows the 3D printed frame on the HoloLens and how the quantitative experiments were performed. The images on the right show, in the upper, the holographic tip at a distance from NDI’s pointer, and in the lower, the position of the Polaris in HoloLens Camera coordinates

For the clinical qualitative assessment (“Accuracy evaluation method” section), registration was also performed rigidly through an application developed in Unity 2018.3 for HoloLens, with which a user can select positions on the directly the hologram and then sample them using the optically tracked and calibrated pointer tip (NDI Tool pointer 8700340, visible in Fig. 2). For the patient phantom, registration was performed twice by the surgeon. One registration was conducted for the patient’s hip (which was successively spatially anchored in situ) and a second registration was performed for the femur. The amount of time necessary for the registration was in total around 3 minutes. Registration was performed through singular value decomposition using seven target positions (similarly to [10]). The targets used for the patient phantom are metallic washers (six millimetres of diameter, also shown in [10]), glued to the patient phantom, which were visible in the CT scan and were segmented and clustered using fuzzy means classification. The metallic washers are replaceable by any hypo- or hyperintense markers, visible in CT (e.g. electrocardiogram patches). Once registration of the models ($T_I^P$) has been computed, every user wearing a HoloLens with optical markers is able to visualise the dynamic MR. (It does therefore not need to be repeated for each user.)

The same procedure can be applied on patients without the washers using anatomical landmarks: greater trochanter, the spina iliaca anterior superior on the pelvis or the medial and lateral epicondyle of the distal femur. These positions can easily be selected by the orthopaedic surgeon in the hologram (or pre-operatively on the CT scan or 3D model), and then can be sampled with any optically tracked instrument while the patient is on the operation table. Transformation $T^I_{\mathrm{P}}$, in Eq. 1, is the result to image-to-patient registration.

To dynamically update the position of the MR while the HoloLens or the patient is moved during diagnostics, the following equation (according to [10]) was used:

1 $$\begin{aligned} T^{C}_{I} = T^{C}_{M} \cdot T^M_{\mathrm{O}} \cdot T^O_{P} \cdot T^P_{I} \end{aligned}$$

In which, *O* is the coordinate system for the OTS, *M* for the HoloLens optical markers, *I* is the image coordinate space, *C* is the HoloLens camera, *P* is the origin of the patient space coordinates (in our study, the OTS origin was used as global reference) and *C* is the camera pose. Through image-to-patient registration and the reference frame on the patient, the holographic model can be aligned to the patient’s body and dynamically updated while moving the limb by changing $T^O_{\mathrm{P}}$, as shown in Fig. 1. $T^M_{\mathrm{C}}$ is computed through hand-eye camera calibration with the additional transformation to left-handed coordinates required by Unity, whereas $T^O_{\mathrm{P}}$ and $T^M_{\mathrm{O}}$ are provided by tracking respectively the markerplate on the patient and on that on the HoloLens camera. Finally, $T^P_I$ represents the image-to-patient transformation matrix computed by the methods previously described (either using a optical pointer to sample anatomical locations, or using an optical markerplate visible in the CT scan).

### Accuracy evaluation method

All experiments were conducted in the OR with constant lighting conditions. The evaluation of the accuracy was performed throughout two experiments and the qualitative clinical assessment. Each user, prior to the experiment, performed Inter-Pupilar-Distance (IPD) calibration and performed calibration of the Hololens to the OTS. The users were requested to move a holographic model at the end of the optically tracked pointer into target positions (as shown in Fig. 2).

The measure of accuracy for the navigation tool, evaluated throughout these studies, was the difference between the position of the optically tracked tool tip (with an offset from the tip, to remove bias due to a physical counterpart of the tool tip) and the target position of the phantom, after the user has moved its holographic equivalent into the target the phantom target position. This error allows to include not only visualisation errors inherent to MR (which are evaluated in [5, 11]) but also the introduction of optical tracking inaccuracy and hand-eye calibration errors [7].

#### Validation phantom

The first experiment was performed on a custom-built validation phantom based on the optical assessment standard ASTM F2554-10 [7]. The phantom presents a total of 28 titanium targets with a divot hole with a diameter of one millimetre on six different orientation planes (as shown in Fig. 2). The optical pointer was calibrated with a transformation at a distance from the tip of the pointer (to avoid the user from positioning the physical tip in the target instead of the holographic tip, as shown in Fig. 2). The holographic tip at the end of the pointer was represented by a green disk-shaped hologram with a red 1-mm spherical centroid, visible in Fig. 2. The user inserted the holographic tip in each of the 28 divot holes of the phantom. The distance between the position at which the holograms were placed with respects to the physical position of the divot hole is what is commonly referred to as visualisation error. The ground truth positions for the divot holes are based on the accurately measured positions with linear sensors ($\upmu$m precision) on the optical validation phantom, which are registered to the OTS through the optical reference frame rigidly connected to it (shown in Fig. 2). Five different users performed the experiment, twice per user.

#### Patient phantom

The second experiment for accuracy evaluation reproduced the Validation phantom on the Patient phantom: the users performed a test by to inserting in each of the 23 metallic washers, a holographic model of the washer (which was set at the same offset to the same tool used in the previous Validation phantom experiment, as shown in Fig. 2). A difference with respect to the Validation phantom was that to acquire ground truth positions for the metallic washers, the position and orientation of the optical reference frame 8700339 were rigidly connected to the patient phantom during the CT acquisition and then extracted through segmentation from the CT scan. The positions of the washers were also segmented from the same CT and the orientation and position of the optical reference frame were used to perform registration from the CT to the OTS (Transformation $T_I^P$). The reason for which this test was conducted is that the positions are spread randomly across the full regions of interest for clinical use of this navigation tool (the femur and the pelvic region of the patient), to simulate the accuracy in evaluating positions across the whole volume of interest for a patient. However, the patient phantom does not have precisely measured positions as targets: the patient’s target positions were metallic washers, previously described in “Image-to-patient registration” section, which were segmented and clustered from the CT scan.

For this reason, the ground truth positions for the 23 metallic washers in the coordinate system of the OTS are not as accurate as the divot holes of the *Validation phantom* (as described in [10]). Furthermore, since transformation $T^O_I$ is not a measured transformation (as per the *Validation phantom*), to compute the ground truth positions of the metallic washers in optical tracking system coordinates (O), the patient optical marker-plate spheres and pins were clustered from the CT scan, and were used to compute $T^O_I$. This process introduces more inaccuracy in the ground truth positions, and therefore a larger error, but should produce comparable results in terms of accuracy with respects to the Validation phantom study.

#### Qualitative assessment

A final evaluation of the navigation tool was performed by an orthopaedic surgeon to reproduce a visual assessment of the navigation tool according to the diagnostic and surgical needs. A CT scan of the patient phantom was performed and through image-to-patient registration using seven metallic washers, the surgeon was able to visualise the segmented hip and femur structures overlaid on the patient (as shown in Fig. 1). Moreover, to avoid any movement of the hip, the patient phantom was strapped to the operating table as can be performed in surgical scenarios (see Fig. 1). Through the use of the reference frame strapped on the phantom’s femur (close to the pelvic region as in Fig. 1), the surgeon was able to visualise the structures move together with the patient’s leg. The surgeon then proceeded with the diagnostic evaluation test, by rotating, abducting and adducting the lower limb, as done in clinical practice for diagnostic evaluations. The femur head was moving according to the updated transformations ($T^O_{\mathrm{P}}$), which allowed him to observe the motion and impingement of the femoral head with the pelvic acetabulum in almost real time (at around 40 Hz update rate). The surgeon then described what was possible to be visualised and what were the limitations and inaccuracies he perceived with the navigation tool, in order to improve the technology according to the needs for the clinic.

## Results

### Validation phantom

**Fig. 3:**
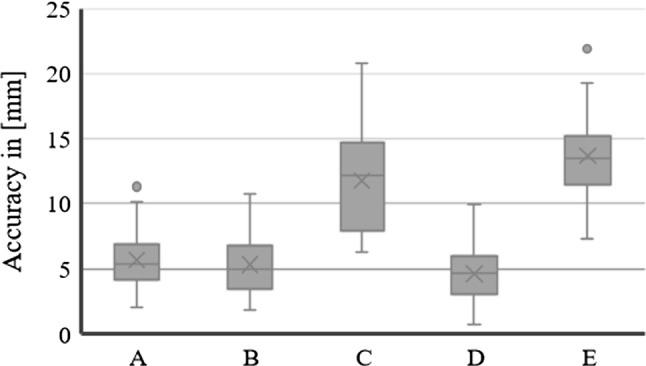
Boxplot of experimental results for the Validation Phantom. Each user is represented by a letter from A to E. Each boxplot represents the errors (with mean, median and standard deviation) across the 28 titanium positions in the Validation phantom for each of the two trials

Five users performed two rounds of the experiment, performing hand-eye calibration and spatial anchoring of the position of the OTS for each round. Twenty-eight target positions, at six different orientation planes, were used in each experiment to evaluate the accuracy of the MR navigation tool. The resulting accuracy in tracking a location at the tip of the instrument in holographic space presented an average $\mu = 8.22$ mm, with standard deviation $\sigma = 2.27$ mm. The results for each user (indicated from A to E) are represented as a boxplot in Fig. 3 and presented in Table 1. Each result is coupled with its mean, standard deviation, coefficient of variation and maximum error for each trial.

**Table 1:** Results for quantitative experiment on validation phantom in (mm)

Users	Mean	SD	Coeff. var. (%)	Max error
A	5.52	2.03	36.86	11.32
	5.87	2.23	38.01	10.03
B	5.12	2.40	46.91	10.73
	5.56	1.83	33.00	9.62
C	9.16	3.53	38.51	20.85
	14.41	1.96	13.62	19.99
D	4.26	1.93	45.24	7.88
	5.01	1.79	35.71	9.91
E	12.51	2.58	20.62	19.28
	14.84	2.43	16.38	21.89
Average	8.22	2.27	32.49	14.15

A One-way ANOVA statistical analysis was run in SPSS 25.0 for each experiment to understand whether there were statistically significant differences in accuracies between users. For the Validation phantom, LSD and Bonferroni post hoc analyses between users C and E $(11.78 \pm 3.19$ mm and $13.67 \pm 2.79$ mm, respectively) as compared to users A, B, D ($5.69 \pm 2.16$ mm, $5.33 \pm 2.16$ mm and $4.63 \pm 2.78$ mm, respectively) deemed statistically significant results ($p = 5.7609\hbox {E}^{-26}$, 2.086$\hbox {E}^{-28}$, 2.6497$\hbox {E}^{-33}$) and also between C and E, statistically significant differences were found (*p* = 0.002418) indicating a significant difference between accuracy within users.

### Patient phantom

**Fig. 4:**
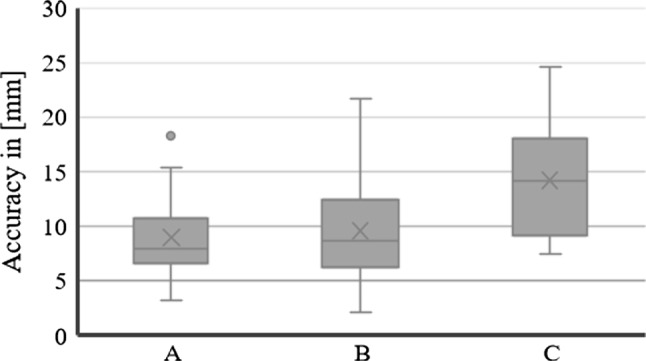
Boxplot of experimental results for the Patient Phantom. Each user is represented by a letter from A to C. Each boxplot represents the errors (with mean, median and standard deviation) across the 23 metallic washer positions in the Patient phantom for each of the two trials

Three users performed two rounds of the experimental protocol for the patient phantom study. A total of 23 target metallic washers were attached to the patient phantom and used to assess the accuracy of locating the tip of the instrument as a hologram. The resulting accuracy was on average $\mu = 10.89$ mm, with $\sigma = 3.78$ mm. The results for each user (indicated from A to C) are represented as a boxplot in Fig. 4 and summarised in Table 2.

**Table 2:** Results for quantitative experiment on patient phantom in (mm)

Users	Average	SD	Coeff. var. (%)	Max error
A	10.58	3.90	36.82	18.96
	7.34	2.06	28.04	11.17
B	8.71	3.25	37.38	14.23
	10.39	5.26	50.61	21.68
C	16.04	4.50	28.07	24.59
	12.29	3.71	30.19	21.15
Average	10.89	3.78	35.19	18.63

With respects to the Patient phantom study, a One-way ANOVA was also performed, and LSD and Bonferroni post hoc analyses found significant differences ($\textit{p} =8.8814\hbox {E}^{-8}$ and $\textit{p} =0.000002)$ between user C ($11.78 \pm 4.58$ mm) when compared to users A and B ($8.96 \pm 3.55$ mm and $9.55 \pm 4.5$ mm, respectively).

### Qualitative assessment

In general, the orthopaedic surgeon provided very positive feedback to the navigation tool. The impression with regard to the visual quality of the holograms was outstanding, and gave an impression of possessing “X-ray” vision. The 3D spatial quality was very good, and provided a life-like impression of the anatomy. Upon movement of the hip joint, a slight lag in the hologram was observed in abrupt movements. There was a slight overlap between the holograms of the femoral head and the acetabulum during hip abduction. Hip rotation gave an excellent impression of actual hip rotation. All spatial views gave equally good visual impression of the hologram. The surgeon noticed a decrease in the registration accuracy during the manipulation of the limb at locations further away from the optical reference frame, which however was not important as long as the frame is strapped to the patient close to locations of interest, as to diminish target registration error.

## Discussion

Results show that the accuracy on average is approximately 8–10 mm for the mixed reality navigation tool. As expected, small increase in the inaccuracy is visible in the patient phantom, which is due to the inaccuracy of the ground truth positions and the larger volume. The accuracy evaluated in this study included calibration inaccuracies in IPD computation, visualisation errors (ability of positioning a holographic model on top of physical correspondent positions [11]), jitter errors due to inertial sensors in the HoloLens (which causes the holograms to slightly shift when the head moves), optical tracking inaccuracies, pivot calibration error to the tip of the optical pointer, camera and hand-eye calibration inaccuracies, calibration error of the position of the OTS with respects to the HoloLens camera (which was repeated for each experiment), segmentation errors in the CT scan, and registration inaccuracies in the phantoms to acquire ground truth. However, it is of great importance that the holograms of the femur and acetabulum is accurately aligned in all joint movements to secure optimal diagnostic conditions. Following discussions with orthopaedic surgeons, in order to lower the inaccuracy due to motion of the pelvis not taken into consideration, two registration processes will be performed: an initial registration process solely for the pelvis and a second for the femur. Moreover, the pelvis will be spatially anchored and measures will be taken to identify the centre of rotation in the femoral head, align this with the acetabulum, possibly with a spatial anchor for translation, as to provide a more realistic reproduction of the joint motion. These separated registration processes could increase or reduce the accuracy of the registration, depending on the landmarks used for each bone.

The statistical analyses deemed significant differences between the users for both the Validation and the Patient phantoms. This means that factors specific to each user (IPD or visualisation error (vergence-accommodation conflict), which is the error committed by visualising both holographic and physical object in the same space [11]) and factors which were repeated for each trial (calibration of the position of the OTS with respects to HoloLens virtual space) can affect the accuracy of the navigation tool. We believe this can be prevented with a better IPD calibration procedure, possibly repeated multiple iterations. More importantly, a multiple posed calibration procedure should be performed when synching the position of the OTS in physical and HoloLens virtual coordinates, not through a single pose, as is currently done. Finally, we believe that a more accurate hand-eye calibration plate may also improve the overall accuracy of the navigation tool.

Overall, considering all the error sources [12]: optical tracking systems introduce approximately around 1.5–2 mm of error [7], hand-eye calibration could also introduce approximately 1–2 mm of error, pivot calibration introduced 0.8 mm, registration, camera and OTS localisation calibrations, IPD calibration, the accuracy of the navigation tool presents promising results for diagnostic assessment, but not as of yet for surgical procedures (where an accuracy inferior to five millimetres is generally acceptable [2]). The errors due to user inaccuracy or physical correspondent positions [11] may be reduced through better visualisation capabilities in HoloLens 2. In surgical scenarios, the accuracy will improve considerably with the trackers placed directly on the bone, rather than the skin for the diagnostic case. However, with suitable trackers, we believe that suitable leg markerplates would reduce relative motion between bones and skin, and since bones do not deform greatly, as compared to soft-tissue [2], rigid registration is suitable for this clinical field. Improvements and testing of multiple leg tracking solutions will be explored in future studies.

With comparison to other studies on HoloLens accuracy for surgical applications, according to [11], visualisation errors introduce more than five millimetres of inaccuracy, however, other studies [5, 13, 14] report up around 2 millimetres. The result reported in [11] could be due to the study setup which introduces registration error through the pose estimation performed on the Vuforia image, which was unaccounted for in the study and may have caused the increase in visualisation error for the holographic study. However, as compared to [5, 11], our studies also present higher inaccuracies, which are due to multiple error sources which are not inspected in other applications (such as, errors due to optical tracking, hand-eye calibration, camera calibration, HoloLens calibration and reconstruction inaccuracies and jitter at various orientations of the HoloLens and at various positions in the OR setting on the entire volume of interest for orthopaedic lower limb surgery). Moreover, the solution presented in this study is usable in clinical settings throughout the whole duration of the diagnostic evaluation, unlike solutions presented in the literature, such as [5, 13] that can currently only work if the distance to the position of interest is up to 20 cm [5] due to inaccurate tracking of AR fiducials, and only with specific lighting conditions. In comparison to other studies performed on phantoms [14], we provide solutions to instrument tracking incorporation and dynamic updates of MR. This study also incorporates the user errors, similarly to [14] in the loose-fit experiment, however, with a different process, due to MR visualisation, which greatly increase the inaccuracy computed. Nevertheless, based on the studies we performed, an accuracy of eight to ten millimetres is approximately expectable as total error with the current solution, which we think can be reduced to five millimetres or smaller with the improvements described previously. Furthermore, in future studies, solutions based on surface reconstruction (stereo-cameras or RGBD cameras) for registration tasks will be explore. These might provide interesting and possibly accurate algorithms to improve the accuracy of this system.

To our knowledge, our paper is the first study in which HoloLens is used in combination with optical tracking systems with applications specific to orthopaedics diagnostics and surgical navigation. This allows the MR navigation tool to be much more flexible and also allows rapid preparation for surgery with sterilisable equipment with comparison to what is available, to our knowledge, in other systems presented in the literature. Moreta-Martinez [5] introduce an orthopaedics surgical navigation tool without OTS (hence without the possibility of instrument tracking), which relies on a 3D-printed bone clamp tracked using the HoloLens camera. The reported errors in this study does not account for registration nor errors in tracking of the surgical 3D-printed bone clamp. Moreover, since this tool relies on the HoloLens to perform pose estimation, poor lighting conditions might interrupt tracking and even cause large inaccuracies in the MR. Furthermore, the tool is patient-specific, meaning that a patient-specific 3D plate must be produced for each clinical case. Our navigation tool can be used in any surgical procedure, without the need to design patient-specific clamps. Moreover, this navigation tool can also display with almost real-time (40 Hz frame update) dynamic holographic updates based on manipulations of the patient limbs.

The ability to perform a real-time examination of the hip joint and pelvis will allow the orthopaedic surgeon to observe the interaction between the femoral head and the acetabulum, thus allowing to accurately diagnose impingement and other anomalies in the hip joint which currently is not possible with conventional static imaging techniques. Holographic imaging during surgical planning will allow for better understanding of the anatomy and thus increase safety and accuracy of the preoperative planning. During surgery, the ability to see the entire skeletal anatomy precisely superimposed on the patient’s body will increase surgical accuracy and allow the surgeon to use smaller incisions to access the specific skeletal anatomy in question.

Overall, in terms of future clinical implications, this new tool has the potential to improve diagnostic accuracy and which can lead to safer, more precise and faster surgeries. It can also to provide better learning conditions for orthopaedic surgeons in training.
